# Oncolytic virotherapy provides a potent therapy option for squamous bladder cancer

**DOI:** 10.1038/s41598-025-96419-3

**Published:** 2025-04-18

**Authors:** Julia Pannhausen, Julia Wirtz, Klaus Mantwill, Per-Sonne Holm, Kristina Schwamborn, Danny D. Jonigk, Jürgen E. Gschwend, Michael Rose, Nadine T. Gaisa, Roman Nawroth

**Affiliations:** 1 Institute of Pathology, Uniklinik RWTH Aachen, 52074 Aachen, Germany; 2 Center for Integrated Oncology Aachen Bonn Cologne Duesseldorf (CIO ABCD), 52074 Aachen, Germany; 3 Department of Urology, Klinikum rechts der Isar, Technical University of Munich, 81675 Munich, Germany; 4 Department of Oral and Maxillofacial Surgery, Medical University of Innsbruck, 6020 Innsbruck, Austria; 5 XVir Therapeutics GmbH, 80335 Munich, Germany; 6 Institute of Pathology, School of Medicine, Technical University of Munich, 81675 Munich, Germany; 7 German Center for Lung Research, DZL, BREATH, 30625 Hanover, Germany; 8 Institute of Pathology, University Hospital, University of Ulm, 89081 Ulm, Germany

**Keywords:** Bladder cancer, Oncolytic virus, Squamous cell carcinoma, Virotherapy, XVir-N-31, YB-1, Bladder cancer, Bladder cancer, Cancer therapy

## Abstract

Prognosis for squamous cell carcinoma (SCC) of the bladder is limited mostly because of lack of effective treatment regimens. Oncolytic virotherapy represents a promising option for bladder cancer and received in 2024 FDA therapy designation for the treatment of non-invasive high-grade bladder cancer (BLCA). For muscle-invasive bladder cancer (MIBC), preclinical studies demonstrated high efficacy of the oncolytic adenovirus XVir-N-31 in urothelial carcinoma (UC). We analyzed the potency of XVir-N-31 virotherapy as a novel treatment option in SCC. Replication of XVir-N-31 has been described to be facilitated by high expression level of Y-Box binding protein 1 (YB-1). Increased *YB-1*-mRNA expression was detected in basal/squamous subtype in TCGA BLCA cohort compared to urothelial and luminal BLCA and correlated with patient outcomes. Furthermore, immunohistochemical staining of 89 SCC on a tissue microarray confirmed strong YB-1 expression in squamous BLCA (sq-BLCA). *In vitro*, XVir-N-31 showed in subtype-specific cell cultures high rates of infection, replication and cell-killing capacity. In a novel *in ovo* xenograft model, XVir-N-31 impaired growth of xenografts of patient-derived *ex vivo* cell lines (p-SCC, p-UC) with growth suppression rates of 39–49%. We provide preclinical evidence *ex vivo* and *in ovo* for high efficacy of XVir-N-31 based oncolytic virotherapy as novel SCC therapy.

## Introduction

Muscle-invasive bladder cancer (MIBC), a prevalent and aggressive urogenital malignant disease, includes various histological subtypes^1,2^. Each subtype exhibits distinct molecular characteristics known to impact phenotypes, prognosis and responsiveness to treatment. Pure squamous cell carcinoma (SCC) represents a rare subtype of muscle-invasive bladder cancer (MIBC). The group of squamous bladder cancers (sq-BLCA) comprising both mixed (urothelial carcinomas with substantial squamous histology) and pure SCC, represents a significant proportion of all diagnosed bladder cancers^3^. While for UC a wide range of approved treatment options such as chemotherapy, immune checkpoint inhibitors^4^ or antibody-drug conjugates^5^ have been developed over the last years, effective therapies beyond radical cystectomy for rare subtypes like SCC are still limited. Squamous differentiation of cancer cells has been associated with poor response to chemotherapy in an adjuvant^6^ as well as neo-adjuvant setting^7^. Thus, there is lack of an effective first-line treatment strategies for squamous bladder cancer^8^.

Recently, the use of oncolytic viruses evolved as a highly effective treatment strategy for BLCA^9,10^. Oncolytic virotherapy utilizes genetically engineered viruses that specifically replicate in cancer cells^9–11^. Consequently, upon viral maturation the cells undergo a lysis process that is characterized by two aspects. First, the cells are driven in an immunogenic cell death characterized by releasing for example damage- and pathogen-associated molecular patterns (DAMPs and PAMPs) and attract the immune system to the tumor site^11,12^. At the same time, lysis of the tumor cells release tumor associated antigens, fostering a specific and systemic anti-tumor immune response. Thus, the molecular mechanism underlying oncolytic virotherapy completely differs from conventional treatment strategies.

Adenoviruses have emerged as one of the most promising candidates in the field of oncolytic virotherapy due to their versatility in genetic manipulation, ease of large-scale production, low toxicity, high genetic stability, and overall safety profile^11^. In 2022, the FDA approved the first adenoviral vector-based gene therapy (Nadofaragene firadenovec, rAd-IFNa/Syn3) for high-risk, non-muscle-invasive bladder cancer (NMIBC) unresponsive to Bacillus Calmette-Guérin therapy^9^. In January 2024, another oncolytic adenoviral construct, cretostimogene grenadenorepvec (CG0070), has been granted breakthrough and fast track status for its FDA approval for treatment of NMIBC^10^.

In urothelial MIBC, efficacy of the oncolytic adenovirus XVir-N-31 (Ad-Delo3-RGD) was demonstrated *in vitro* and *in vivo* using a humanized murine model by our group^12–14^. Tumor-selective replication of XVir-N-31 has been achieved through several genetic modifications^15^. These modifications include deletion of the viral E1A13S and E1B19k gene products which are predominantly involved in the transactivation of transcription factors to viral promoter structures such as the E2 region, encoding for the viral polymerase^16^. One of these factors is the protein Y-Box binding protein 1 (YB-1). Beyond its role as a regulator of gene transcription and expression, it is also involved in splicing, translation, and cell proliferation^17^ and is upregulated in multiple tumor entities, including MIBCs, where it is associated with poor prognosis^18^. YB-1 is predominantly localized in the cytoplasm but can be translocated into the nucleus^19^ e.g. after cellular stress or upon viral infection^15^. Our group has demonstrated that YB-1 binds directly to the E2-Late promoter and increased expression level of YB-1 dramatically improves replication of XVir-N-31 and, importantly, results even in a partial rescue of a non-replicating E1 deleted adenovirus. In cells, treated with siRNAs directed against YB-1, adenoviral replication is significantly downregulated^16,19–24^. However, in SCC YB-1 expression levels have not been thoroughly examined yet.

Therefore, we aimed to examine whether the oncolytic adenoviruses XVir-N-31 might be a suitable novel treatment strategy in SCC. We analyzed YB-1 expression and its subcellular localization in patient tissue samples using a German-wide cohort comprising 89 cases of confirmed sq-BLCA. In addition, we examined therapeutic efficacy of XVir-N-31 in established urothelial and sq-BLCA cell lines, as well as in patient-derived *ex vivo* cell culture models, and developed an *in ovo* model to evaluate the effects of XVir-N-31 on three-dimensional tumor xenografts.

## Results

### YB-1 expression in BLCA subtypes and associations with patients’ outcome

**Fig. 1:**
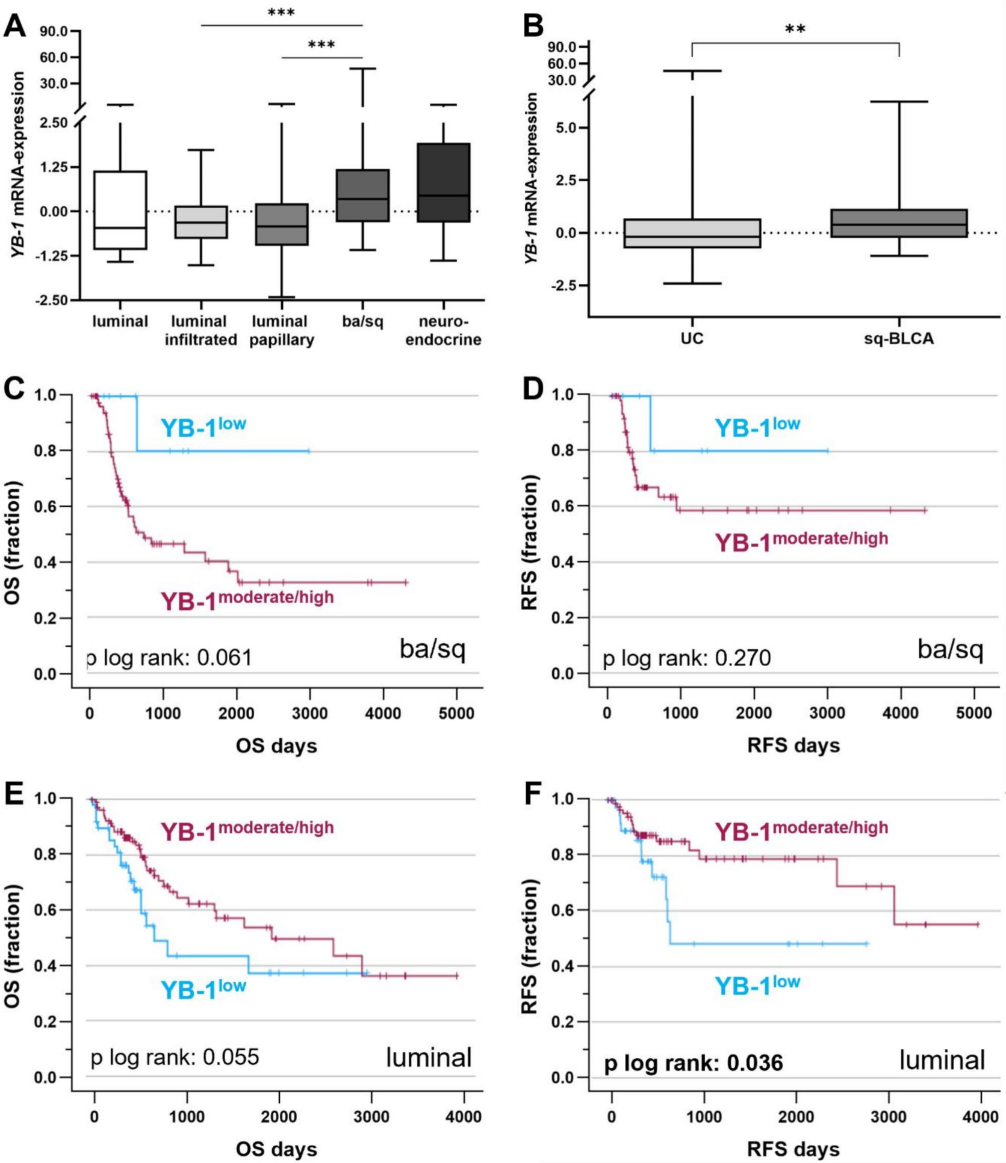
*YB-1* mRNA expression in BLCA subtypes predicts patients’ outcome. (**A**,**B**) Independent BLCA data sets of the TCGA network, with (**A**) *YB-1* mRNA expression classified by molecular subtypes: luminal (*n* = 23), luminal-infiltrated (*n* = 58), luminal-papillary (*n* = 110), basal/squamous (ba/sq) (*n* = 108) and neuroendocrine (*n* = 16). ***P < 0.001 (Kruskal–Wallis and Dunn’s multiple comparison tests). (**B**) Histological subtyping of TCGA data confirmed high *YB-1* mRNA expression in sq-BLCA (*n* = 44) compared to UC (*n* = 271). **P < 0.01 (Mann–Whitney U test). (**C**–**F**) Kaplan–Meier survival curves illustrate (**C**,**E**) overall survival (OS) and (**D**,**F**) relapse-free survival (RFS) of BLCA patients with moderate to high *YB-1* mRNA expression (red curve) compared to low *YB-1* mRNA expression (blue curve) classified by molecular subtypes: (**C**,**D**) basal/squamous-like (ba/sq) (*n* = 98) (**E**,**F**) and luminal type (*n* = 164) based on TCGA BLCA data sets. YB-1^moderate/high^ in basal/squamous UC with mean OS 1832.7 days ± 251.0, 95% CI 1340.9–2324.6 and mean RFS 2659.1 days ± 303.9, 95% CI 2063.5–3254.8. YB-1^low^ in basal/squamous UC with mean OS 2495.8 days ± 418.8, 95% CI 1675.0–3316.6 and mean RFS 2482.2 days ± 430.9, 95% CI 1637.6–3326.8. YB-1^moderate/high^ in luminal UC with mean OS 2219.1 days ± 221.6, 95% CI 17845.0–2653.5 and mean RFS 2962.9 days ± 242.0, 95% CI 2488.6–3437.1. YB-1^low^ in luminal UC with mean OS 1467.1 days ± 242.1, 95% CI 992.6–1941.7 and mean RFS 1581.3 days ± 287.2, 95% CI 1018.5–2144.2. For all illustrated box plots: Horizontal lines: grouped medians. Boxes: 25–75% quartiles. Vertical lines: range, maximum and minimum.

We first analyzed expression level of YB-1 as a molecular parameter supporting replication and therapeutic efficacy of XVir-N-31 in BLCA subtypes. Utilizing the independent BLCA dataset from The Cancer Genome Atlas (TCGA), YB-1 expression was detected across molecular subtypes defined by Kamoun et al.^1^. *YB-1* mRNA expression level was significantly higher in basal/squamous (ba/sq) UC tumors (*n* = 108) compared to luminal-infiltrated (*n* = 58) and luminal-papillary UC (*n* = 110) (*p* < 0.001) (Fig. 1A). The neuroendocrine subtype (*n* = 15) exhibited *YB-1* mRNA expression levels comparable to those in basal/squamous UC tumors.

Using the TCGA BLCA dataset by Robertson et al.^2^, sq-BLCA (*n* = 44) showed significantly elevated YB-1 expression (fold change = 0.375) compared to UC (*n* = 271) (*p* < 0.01) (Fig. 1B). Additionally, 34.1% of sq-BLCA patients exhibited high YB-1 expression (fold change > 1.0) in contrast to 19.1% of UC patients. These results might indicate that SCCs are particularly suitable for supporting potent replication and thus treatment success with XVir-N-31.

To assess overall survival (OS) and relapse-free survival (RFS) rates using univariate Kaplan-Meier survival curves, TCGA BLCA expression data was dichotomized into tumors with low and moderate to high YB-1 expression level based on the lower 25% quartile cutoff. In summary, basal/squamous UC patients with moderate to high YB-1 expression tended to shorter OS (mean: 1832.7 days) compared to those with low YB-1 expression (mean: 2495.8 days) (*p* = 0.061) (Fig. 1C). A similar trend was observed for RFS (Fig. 1D). Notably, only 11% of basal/squamous UC patients had low YB-1 expression, compared to 89% with high YB-1 expression. Conversely, luminal UC patients with increased YB-1 expression were associated with a trend towards longer OS (mean: 2219.1 days) compared to those with low YB-1 expression with lower OS (mean: 1467.1 days) (*p* = 0.055) (Fig. 1E). This trend was supported by significantly longer RFS in luminal UC patients with strong YB-1 expression (mean: 2962.9 days) compared to those with low YB-1 expression (mean: 1581.3 days) (*p* = 0.036) (Fig. 1F).

**Fig. 2:**
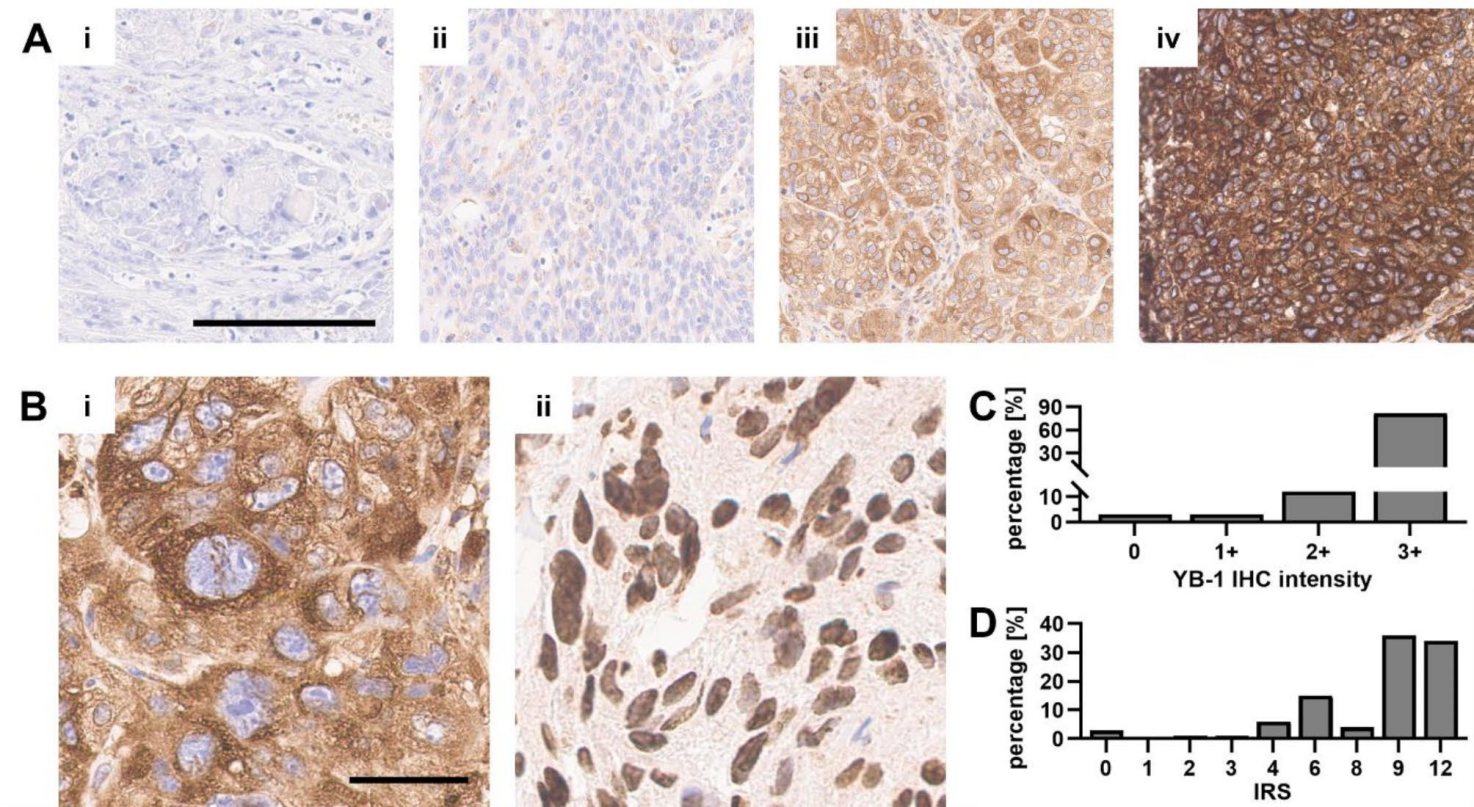
YB-1 expression and localization in sq-BLCA patient samples. (**A**–**B**) Representative images of immunohistochemically YB-1 staining in sq-BLCA patient tissues: (**A**) (**i**) negative YB-1 protein expression (**ii**) low/minimal YB-1 protein expression (intensity + 1), (**iii**) moderate/intermediate YB-1 protein expression (intensity + 2), (**iv**) high/strong YB-1 staining (intensity + 3). Scale bar 100 μm. (**B**) (**i**) YB-1 staining of nucleus and (**ii**) YB-1 staining of cytoplasm. Scale bar 50 μm. (**C**,**D**) YB-1 expression and distribution after IHC staining of sq-BLCA (*n* = 89) FFPE-sections. IHC scoring was estimated by percentage of YB-1 positive cells and (**C**) the intensity of positive staining in corresponding sections of sq-BLCA tumors. (**D**) IRS score was calculated as product of IHC intensity and percentage of positively stained cells according to Remmele and Stegner^25^.

These data were validated immunohistochemically in a Germany-wide cohort of sq-BLCA patient tissue samples (Fig. 2; cohort characteristics are detailed in Supplementary Table S1). Tissue microarrays were evaluated based on staining intensity and the percentage of positive cells, to finally calculate an adapted immunoreactive score (IRS) developed by Remmele and Stegner^25^. YB-1 protein expression was observed in 97% o analyzed sq-BLCA patients based on YB-1 staining intensity (scored 0–3+; Fig. 2Ai–iv) and IRS. At the subcellular level, nuclear YB-1 localization was detected in 1.2% o cases (Fig. 2Bi) while 3.5% exhibited expression in both, the nucleus and cytoplasm. Only cytoplasmic localization was observed in 95.3% o cases (Fig. 2Bii). Intensity analysis showed 82% of sq-BLCA specimens with intensity of 3 + and 34% with IRS of 12 (Fig. 2C, D). Overall, we demonstrated that YB-1 is broadly and highly expressed in this sq-BLCA patient cohort.

Furthermore, clinico-pathological parameters of this sq-BLCA patient cohort were correlated with YB-1 protein levels (Supplementary Table S2). A significant association with tumor grading (*p* = 0.014, Spearman *r* = 0.268) was revealed, indicating that high YB-1 expression is more frequently observed in high-grade tumors (G3-G4), whereas low-grade tumors (G1-G2) show lower YB-1 expression. Additionally, a trend toward significance was observed for lymph node status (*p* = 0.053, Spearman *r* = 0.230), with high YB-1 expression being more common in pN-positive tumors, overall supporting the survival data from the TCGA cohort. In contrast, tumor stage, age, and gender showed no significant associations, indicating that YB-1 expression is independent of these factors.

### Efficacy of YB-1 selective oncolytic virotherapy in SCC and UC cell lines and patient-derived models

**Fig. 3:**
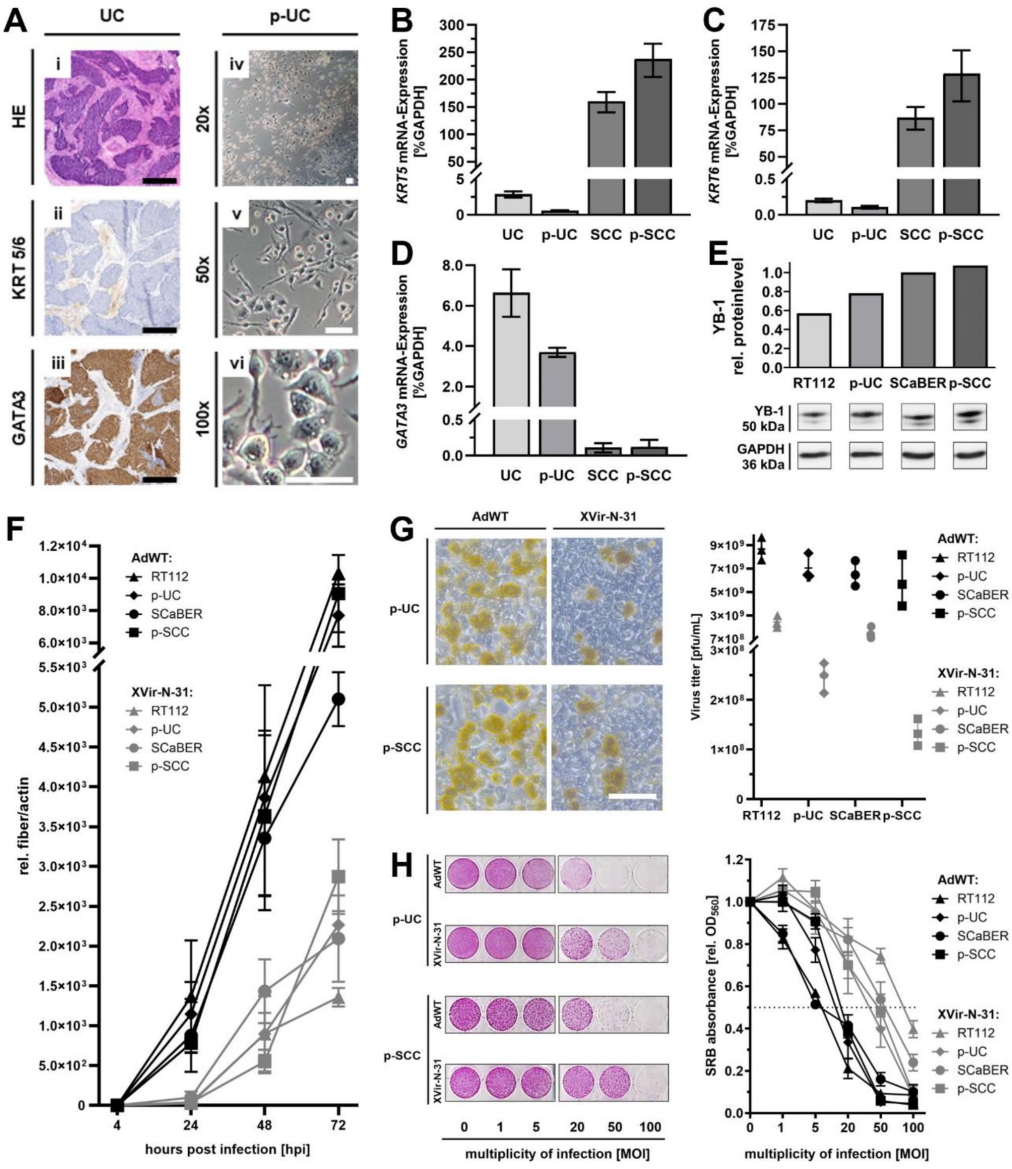
Efficacy of YB-1 selective oncolytic virotherapy in SCC and UC cell culture models. (**A**–**D**) Characterization of patient-derived UC model (p-UC). (**A**) Histological characterization of original UC tissue with (**i**) HE staining, (**ii**) immunohistochemical anti-KRT5/6 staining, (**iii**) immunohistochemical anti-GATA3 staining. p-UC morphology was characterized by light microscopy (**iv**–**vi**) at different magnifications (20×, 50×, 100×). Black scale bars correspond to 500 μm and white to 100 μm. (**B**–**D**) Confirmation of urothelial character of p-UC and squamous-like character of p-SCC with marker expression of (**B**) *KRT5*- (**C**) *KRT6*- and (**D**) *GATA3*-mRNA compared to the original UC and SCC tumor with *GAPDH* as control. (**E**) Overall YB-1 protein level in uninfected BLCA cell lines (RT112, p-UC, SCaBER, p-SCC) was measured by immunoblotting. DOWN: YB-1 (50 kDa) and GAPDH (36 kDa) membranes. The raw immunoblot is presented in (Supplementary Fig. S1). UP: protein bands normalized at SCaBER YB-1 protein level and GAPDH. (**F**–**H**) Viral infection process: (**F**) BLCA cell lines (RT112, p-UC, SCaBER, p-SCC) were infected with 20–100 MOI of indicated viruses and harvested 4, 24, 48 and 72 hpi. After DNA phenol-chloroform extraction viral replication was assessed by qPCR to amplify viral fiber DNA. Values were normalized to the 4 hpi values. (**G**) Hexon titer test with representative staining (left) and detected virus titers (right). BLCA cell lines (RT112, p-UC, SCaBER, p-SCC) cells were infected with 10-200 MOI and harvested with supernatant 72 hpi to quantify formation of infectious particles. (**H**) SRB assay (*n* = 3–6 independent experiments) with representative staining (left) and detected SRB absorbance (right). BLCA cell lines (RT112, p-UC, SCaBER, p-SCC) cells were infected with indicated MOIs and stained with SRB five to six days after infection to measure cell-killing effect.

High YB-1 expression in sq-BLCA specimen prompted us to examine oncolytic virotherapy efficacy *in vitro* using established BLCA cell lines representing SCC and NMIBC (SCaBER and RT112). In addition, we established primary cells derived from patients diagnosed with a SCC (p-SCC), which were previously described^26^, and a newly generated patient-derived urothelial cell line p-UC. Original tumor tissue of p-UC was characterized immunohistochemically for luminal urothelial differentiation (Fig. 3A) by negative KRT5/6 staining (Fig. 3Aii) and strong GATA3 staining (Fig. 3Aiii). The derived p-UC cells displayed a luminal, spindle-like morphology (Fig. 3Aiv–vi). mRNA expression of basal-like markers *KRT5*, *KRT6*, and luminal-urothelial marker *GATA3* confirmed p-UC/UC and p-SCC/SCC classification (Fig. 3B–D). Furthermore, YB-1 protein expression was demonstrated in all cell lines via immunoblotting (Fig. 3E, Supplementary Fig. S1).

Next, we focused on the efficacy of oncolytic virotherapy *in vitro* using the oncolytic virus XVir-N-31 and wild-type AdWT as control. First, we analyzed replication of the virus, monitored 4–72 hpi by qPCR to determine viral genomic copies (*fiber* DNA) and to assess viral gene expression after infection (Fig. 3F). We could demonstrate that both, AdWT and XVir-N-31 show exponential replication kinetics. As expected, AdWT exhibited higher replication rates and viral titers than XVir-N-31 in all cell lines. However, XVir-N-31 demonstrated exponential replication with titers exceeding 1.8 × 10^6 pfu/mL (Fig. 3G). Most importantly, XVir-N-31 showed potent cell-killing capacity across all tested cell lines, with MOI-dependent (multiplicity of infection) effects (Fig. 3H).

### YB-1 translocation into the nucleus visualizes adenovirus replication compartment formation in BLCA cells post infection

**Fig. 4:**
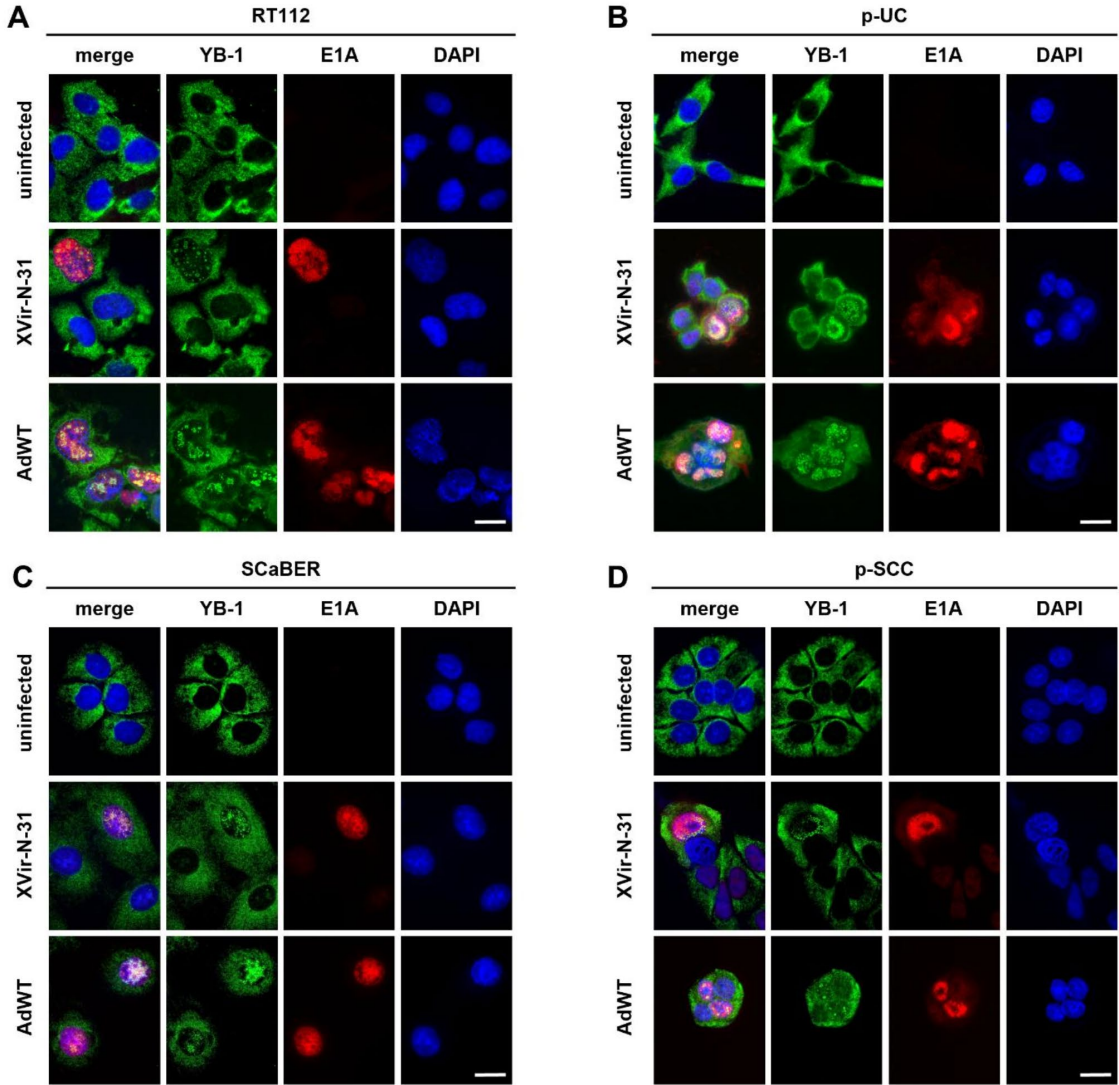
YB-1 translocation and formation of adenovirus replication compartments post infection in BLCA cells. Immunofluorescence staining of YB-1 and E1A to visualize adenovirus replication compartments (AdRC). UC *in vitro* and patient-derived *ex vivo* models (**A**) RT112 and (**B**) p-UC and SCC *in vitro* and patient-derived *ex vivo* models (**C**) SCaBER and (**D**) p-SCC were not infected or infected with the indicated virus. Cells were fixed 48 hpi. E1A staining served as infection control. Scale bar 20 μm.

Adenovirus infection and replication cause extensive reorganization of the host cell nucleus^19^, leading to the formation of adenovirus replication compartments^19,27^ and triggering YB-1 nuclear relocalization^27^. We confirmed this by immunofluorescence staining for YB-1 and the viral protein E1A as a marker for infected cells, in UC (RT112, p-UC; Fig. 4A, B) and SCC models (SCaBER, p-SCC; Fig. 4C, D). At 48 hpi, YB-1 protein translocation into the nucleus was observed exclusively in infected cells, with nuclear YB-1 patterns varying between cell lines potentially reflecting differences in replication efficiency. Taken together, these data showed that adenoviral infection of BLCA cells induced the described YB-1 nuclear translocation and support a role of YB-1 in successful adenoviral replication^16^.

### Oncolytic virus treatment impairs *in ovo* tumor growth of patient-derived SCC and UC BLCA cells

**Fig. 5:**
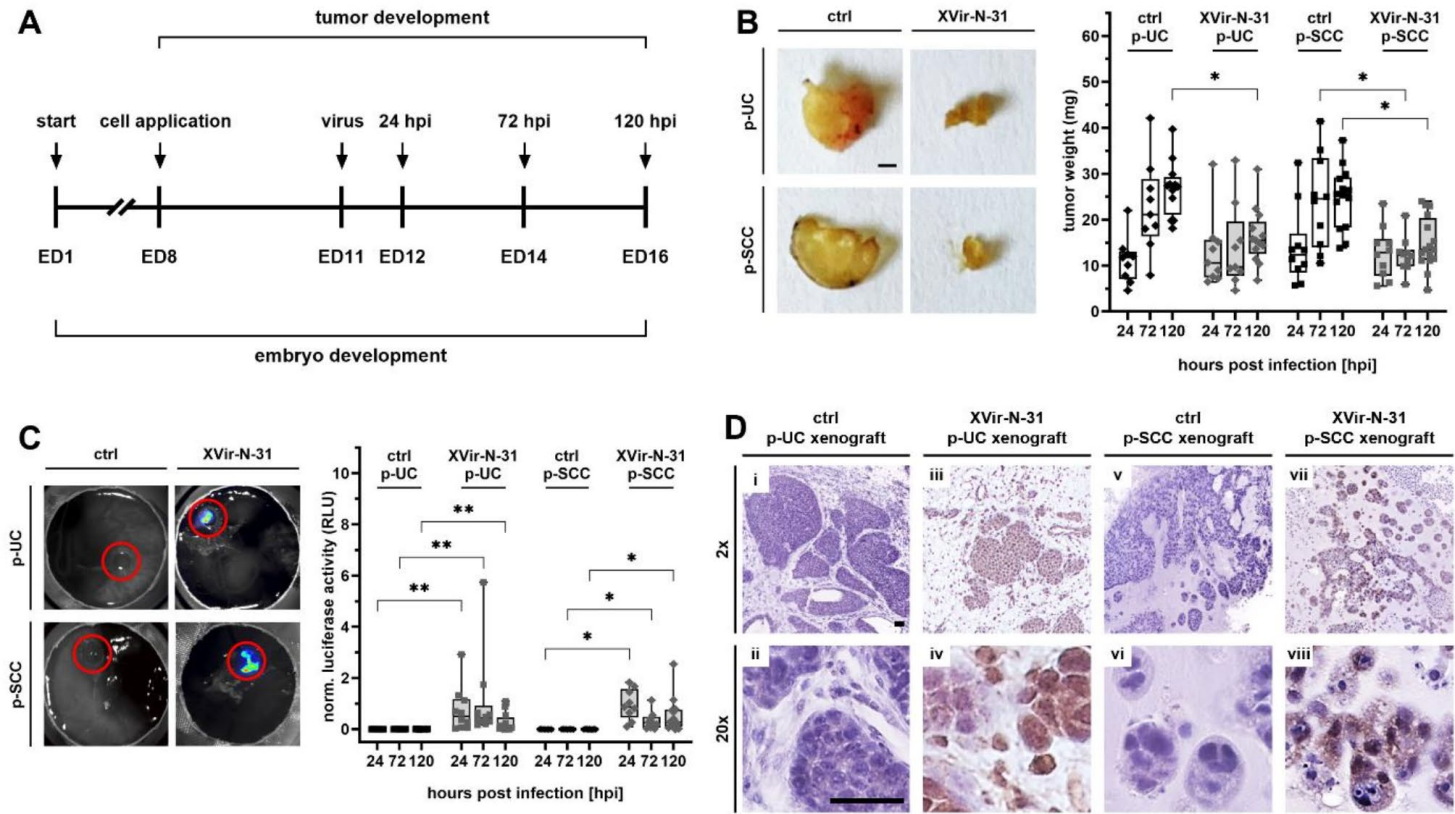
Oncolytic virotherapy suppresses *in ovo* tumor growth of patient-derived BLCA cells. (**A**) Schematic illustration of the experimental timeline. Fertilized eggs were incubated till ED8 to applicate patient-derived cell culture models (p-UC, *n* = 61; p-SCC, *n* = 72). Treatment started at ED11 by adding either XVir-N-31 (127.75 MOI) or H_2_O as control. Xenografts were harvested at ED12 (24 hpi), ED14 (72 hpi) and ED16 (120 hpi) to measure luminescence intensity and tumor weight. (**B**) XVir-N-31 treatment of CAM tumors reduces tumor size. Quantification of tumor weight from harvested CAM tumors derived from p-UC and p-SCC cells treated with H_2_O or XVir-N-31 24, 72 or 120 hpi. H_2_O treatment served as control. *n* = 9–16 for each measurement. *P < 0.05 (Kruskal–Wallis and Dunn’s multiple comparison tests). Scale bar 1000 μm. (**C**) Quantification of luciferase activity by measurement of bioluminescence images (left) taken from CAM tumors derived from p-UC and p-SCC cells treated with H_2_O or XVir-N-31 were harvested 24, 72 or 120 hpi. H_2_O treatment served as control. *n* = 9–16 for each measurement. *P < 0.05, **P < 0.01 (Kruskal–Wallis and Dunn’s multiple comparison tests). (**D**) Immunohistochemistry for Hexon on representative FFPE-sections of p-UC (**i–iv**) and p-SCC (**v–viii**) untreated (**i, ii, v, vi**) and XVir-N-31 treated (**iii, iv, vii, viii**) xenografts harvested 72 hpi after CAM assay. Scale bars correspond to 50 μm (2× and 20× magnification).

We investigated XVir-N-31’s impact on UC and SCC tumor growth using the chicken chorioallantoic membrane (CAM) model (Fig. 5A). This model simulates an immunocompromised three-dimensional *in vivo* system with host-derived vasculature and extracellular matrix^28^. We infected xenografts of patient-derived cell lines p-UC (*n* = 61) and p-SCC (*n* = 72) with a luciferase-expressing XVir-N-31 derivate (XVir-N-1) *in ovo* (Fig. 5B–D). At 120 hpi, infected p-UC and p-SCC xenografts showed significant weight reduction (39% and 49%, respectively) compared to uninfected samples (Fig. 5B). Furthermore, infected xenografts showed significant bioluminescent signals (Fig. 5C) and were exclusively positive for the adenoviral protein Hexon (Fig. 5D). In conclusion, XVir-N-31 infection and replication effectively suppressed *in ovo* tumor growth in patient-derived xenografts of SCC.

## Discussion

In the present study, we demonstrated that application of oncolytic virotherapy is a promising treatment strategy for SCC. Our analysis indicates that in SCC expression level of YB-1 is higher relative to UC which would support oncolytic adenovirus XVir-N-31 based therapy and would make this subtype in particular suitable for this treatment strategy. Thus far, various studies suggested overexpression of YB-1 in bladder cancer (BLCA) in general correlating with poor prognosis^24,29^. We extended these data to sq-BLCA specimen and compared data from our patient tissue samples with the TCGA database. Most importantly, we demonstrated that YB-1 is strongly expressed in a high percentage of squamous bladder cancers. Furthermore, we showed that in BLCA with basal/squamous differentiation, the overexpression of YB-1 is associated with poor prognosis. However, this cannot be generalized to BLCA, as seen in luminal UC with the exact opposite correlation between YB-1 and prognosis, and where less than 70% of luminal UC cases exhibit high YB-1 expression compared to sq-BLCA cohort with 89%. In addition to our focus on sq-BLCA, our TCGA analysis also revealed that the neuroendocrine subtype exhibited *YB-1* mRNA expression levels comparable to those in basal/squamous UC tumors. This suggests that XVir-N-31 virotherapy could also be suitable for this subtype, but must be validated in future studies.

In consistence with data from other cancer entities^17,19,24^, our immunofluorescence analysis localized YB-1 mostly in the cytoplasm of bladder cancer cells. There are only few examples with nuclear protein localization of YB-1, such as ERG-negative prostate cancer, where YB-1 upregulation and nuclear translocation is associated with poor prognosis^30^.

Oncolytic adenovirus-based therapy has been proven to be highly efficient and extremely well tolerated^11^. We tested its efficacy as a novel strategy for therapy in SCC and provided functional evidence that XVir-N-31 can infect, replicate in and induce lysis of sq-BLCA cell lines, derived from primary SCC tissue and established basal/squamous cell lines. Nevertheless, sufficient cell-killing was observed in all tested cell lines. One safety aspect when using oncolytic virotherapy is to avoid a hypothetical induction of a cytokine storm that could be elicited by the virus with severe consequences for the patient^31^. Since the human immune system is perfectly adapted to adenoviral infections, the lower replication rate of XVir-N-31 warrants avoidance of such an immunological response. Our group has also demonstrated that the replication potency of XVir-N-31 is sufficient to induce in combination with cyclin dependent kinases 4/6 inhibitor (CDK4/6i) abscopal anti-tumor effects in a humanized mouse model^32^. As for the role of YB-1 on replication, adenoviral infection caused nuclear shuttling of YB-1 in the* in vitro* and patient-derived cell culture models supporting the described role of YB-1 upon oncolytic adenovirus XVir-N-31 replication^16,23^. Thus, detection of the YB-1 expression level might be useful as a molecular marker for stratification of patients for this therapy.

*In vivo*, our group showed previously growth inhibition in a non-squamous MIBC model of 42% based on an orthotopic mouse model^12^. In this study, we established the CAM model as a three-dimensional tumor model for testing the response to an oncolytic virus. One challenge of this model is the size of the resulting xenografts, which makes a direct intratumoral injection of the virus impossible^33^. We showed that topical application of the virus is sufficient to infect the xenograft. First, we observed a significant reduction in tumor size after XVir-N-31 infection, which resembled the efficacy seen in our immune compromised murine models. Second, in this study we could confirm successful XVir-N-31 replication in infected xenografts. Thus, we established a three-dimensional model that allows replacement of immune compromised animal models when testing effects on tumor growth using an oncolytic virus.

Oncolytic virotherapy is still in the early stage of entering clinical care. Advancements in next generation of viral vectors are promising. For example, XVir-N-31, unlike cretostimogene grenadenorepvec^10^, infects cells independently of Coxsackievirus and adenovirus receptor (CAR) expression and retinoblastoma protein (RB) status^12^. This is important, as CAR expression is downregulated in about 50% of BLC specimens^34 and^, loss of RB is reported in only a subset of BLCA patients, e.g. 20–25% of basal/squamous UC^1^. However, efficacy of adenoviral therapies could further benefit by combining approaches targeting viral regulatory components. Accordingly, our group has shown that combining XVir-N-31 with CDK4/6i enhances its anti-tumor effects by increasing virus production, genome replication, particle formation, and cancer cell-killing, as shown* in vitro* and *in vivo* using humanized murine models^13,14,35^. Future research should address this approach, particularly for the treatment of challenging mixed and pure SCC of the bladder.

In conclusion, we provide strong evidence that the use of an oncolytic adenovirus might provide a completely novel approach for the treatment of BLCA with squamous differentiation. This subtype would be particularly suitable for this therapy due to its high YB-1 expression, essential for successful adenoviral XVir-N-31 replication.

## Methods

### TCGA BLCA data set

Public BLCA data sets from The Cancer Genome Atlas (TCGA)^36^ network, including RNASeqV2 data (level 3) were subclassified as described^1,2^ using the cBio Cancer Genomics Portal (http://cbioportal.org)^37^.

### Patient samples BLCA data set

Our study included 89 patient tissue samples diagnosed with squamous bladder cancer (sq-BLCA) collected by the German Study Group of Bladder Cancer (DFBK e.V.). Sq-BLCA includes 54 pure (100%) squamous cell carcinomas (SCCs) and 35 mixed urothelial carcinomas (UCs) with > 70% squamous differentiation. Supplementary Table S1 summarizes patient characteristics and Supplementary Table S2 the correlation of clinico-pathological parameters (gender, tumor stage, pN status, age) with YB-1 expression levels based on the semiquantitative immunoreactive score (IRS) according to Remmele and Stegner^25^. Additional tissue was obtained from the RWTH centralized biomaterial bank. Patients gave written informed consent and experiments followed the regulations of the RWTH centralized biomaterial bank and the Declaration of Helsinki. This was approved by the Institutional Ethical Review Board of the Medical Faculty of RWTH Aachen University (EK 286/11, EK 206/09, EK 23-058).

### Immunohistochemistry (IHC)

For histological analysis, formalin-fixed, paraffin-embedded (FFPE) sections underwent standard hematoxylin and eosin (HE) staining. IHC with primary antibody anti-Hexon (1:100, AB1056, Chemicon) was performed with Dako EnVision FLEX system kit (K8000; Agilent Dako), DAB + Subtrate System (K3468, Agilent Dako) and haematoxylin for counterstaining as described^26^. IHC staining of anti-GATA3 and anti-KRT5/6 was performed on autostainer 360 (ThermoFisher Scientific) as previously specified^38^. YB-1 IHC staining (1:4000, ab76149, Abcam) was performed on a Leica Bond (Leica) with heat induced epitope retrieval (epitope retrieval solution 1) for 30 min. Expression levels were analyzed using the semiquantitative immunoreactive score (IRS) according to Remmele and Stegner^25^.

### Cell lines, adenoviral vectors and infection

The human bladder cell lines RT112 (NMIBC, urothelial cell carcinoma; RRID: CVCL_1670), SCaBER (MIBC, squamous cell carcinoma, RRID: CVCL_3599) and human embryonic kidney cell line HEK293 (RRID: CVCL_0063) were used. Cells were cultured in DMEM high glucose medium (11965092, ThermoFisher Scientific), supplemented with 10% fetal bovine serum (FBS-11 A, Capricorn Scientific) and 5% LGPS (200 mM L-glutamine, 50 U/ml penicillin, 50 mg/L streptomycin; 10378016, ThermoFisher Scientific), at 37 °C in 5% CO_2_. Tests for possible mycoplasma infections were performed regularly. All cell lines were authenticated by single nucleotide polymorphism (SNP) typing within the last three years and were already available at the Institute of Pathology RWTH Aachen University and the Department of Urology Technical University of Munich. For patient-derived *ex vivo* models, primary cells (p-SCC, p-UC) were isolated and cultured as described^39^. Unless otherwise stated, all cell culture experiments were performed independently at least three times.

The adenoviral construct XVir-N-31, or XVir-N-1, featuring a CMV-luciferase cassette in the XVir-N-31 E3 region, and wild-type human adenovirus serotype 5 (AdWT) with an RGD motif in the fiber knob, were used^15^. After seeding (cell density: 1.0–6.0 × 10^4^ cells/cm^2^) and attachment overnight, cells were infected with virus particles at specified MOI (multiplicity of infection) for 1 h at 37 °C in serum-free medium and supplemented with 1–2 ml medium in total 1 hpi.

### DNA extraction

4-72 hpi, cells were lysed in DNA lysis buffer (10 mM Tris-HCl (pH 8), 100 mM NaCl, 25 mM EDTA (pH 8), 0.5% SDS) and proteinase K overnight at 56 °C and 300 rpm. DNA was extracted with phenol-chloroform-isoamyl alcohol (P2069, Sigma-Aldrich) and precipitated with sodium acetate and 100% ethanol after washing step with 70% ethanol (34852-M, Sigma-Aldrich).

### RNA extraction

For RNA extraction from fresh frozen bladder tissue, tissue was manually microdissected. RNA isolation of fresh frozen bladder tissue was performed using the Maxwell^®^ 16 LEV simplyRNA Tissue Kit (AS1280, Promega) according to the manufacturer’s protocols.

For cell culture RNA isolation, cells were lysed with RNA lysis buffer (740906.125, Macherey Nagel) and RNA isolation was performed using the NucleoSpin^®^ RNA Plus Kit (740984.50, Macherey Nagel) according to manufacturer’s instructions.

For cDNA synthesis the reverse transcription system kit (A3500, Promega) was used with 1 µg of total RNA and Thermal-Cycler C1000 Touch (Bio-Rad). Mastermix components and cycling conditions are described in Supplementary Table S3.

### Quantitative real-time reverse transcription PCR

Expression and viral replication were analyzed by real-time qPCR (RT-qPCR) using CFX96 touch real-time PCR system (Bio-Rad), iQ™ SYBR^®^ Green Supermix (1725125, Bio-Rad) and Power SYBR™ Green PCR Master Mix (4367659, ThermoFisher Scientific). Samples were measured in triplicates. Relative expression was quantified using the 2^-^^∆∆Ct^ method with *GAPDH* or *beta-Actin* serving as reference genes. PCR primer sequences and PCR conditions are described in Supplementary Table S3.

### Immunoblotting

Cells were lysed in RIPA lysis buffer (50 mM Tris, 150 mM NaCl, 10 mM EDTA, 1% IGEPAL, 0.5% sodium deoxycholate, 0.1% SDS) and centrifuged for 30 min at 30,000 × g, 4 °C. Protein concentrations of lysis supernatants were quantified using Pierce™ BCA Protein Assay kit (23225, ThermoFisher). Protein lysates were separated by SDS-PAGE using Mini-PROTEAN Tetra Vertical Electrophoresis Cell (Bio-Rad Laboratories) and transferred to PVDF membranes (10600001, GE-Healthcare) using Mini-TransBlot Electrophoretic Transfer Cell (Bio-Rad Laboratories). Membranes were incubated in 5% w/v nonfat dry milk blocking buffer for 1 h followed by incubation with primary antibodies, anti-YB-1 (1:1000, ab76149, Abcam), anti-GAPDH (1:1000, #2118, Cell Signaling Technology), overnight at 4 °C. Membranes were incubated with secondary antibody, anti-rabbit (1:10000, 711-036-152, Jackson ImmunoResearch) for 1 h. Immunoreactive proteins were visualized using Amersham ECL-reaction (89168-782, GE-Healthcare) and iBright FL1500 imaging system (Bio-Rad).

### Immunofluorescence staining

48 hpi, cells were fixed with ice-cold methanol: acetone (1:1) for 20 min at -21 °C, followed by blocking with 3% BSA (A9418, Sigma-Aldrich) for at least 30 min. Staining was performed with primary antibodies, anti-YB-1 (1:500, ab76149, Abcam), anti-E1A (1:500, M73, Santa Cruz), for 1 h. Coverslips were incubated with fluorescence-coupled secondary antibodies, Alexa488 (1:500, #A-11008, ThermoFisher Scientific), Alexa549 (1:500, #A-11001, ThermoFisher Scientific), for 30 min in the dark. DAPI counterstaining and coverslip mounting was used with ProLong^®^ Gold Antifade (P36930, ThermoFisher Scientific). Images were captured using AxioVert.A1 Microscope (Zeiss) and AxioCam ERc5s Camera (Zeiss).

### Hexon titer test

72 hpi, cells and supernatant were harvested and lysed during three freeze/thaw cycles. Supernatant was added during seeding of HEK293 cells at a density of 1.0 × 10^5^ cells/cm^2^ and infected with 10 and 50 µL of viral supernatant dilutions ranging from 10^− 0^ to 10^− 5^. 48 hpi, cells were fixed with 100% ice-cold methanol (34860, Sigma-Aldrich) for 20 min at 4 °C and washed with 1% BSA-PBS solution. Primary antibody goat anti-Hexon (1:500, AB1056, Chemicon) was incubated for 1 h at 37 °C. Secondary antibody rabbit anti-goat (1:500, P0449, Dako) was incubated for 1 h at 37 °C. DAB + Substrate System (K3468, Dako) was used for visualization for 30 min. Viral infectious titer was calculated as the ratio of the average number of positive cells counted times visual fields, and the added virus volume times the viral dilution factor.

### Sulforhodamine B (SRB) assay

120-144 hpi, cells were fixed with 10% ice-cold trichloroacetic acid (T6399, Sigma-Aldrich) for at least 1 h at 4 °C and washed with tap water. Staining was performed with 0.05% SRB solution for 30 min and excess staining solution was removed by washing with 1% acetic acid. To measure cell-killing efficacy, stained cells were dissolved with 10 mM Tris and absorbance (590 nm) and measured using microplate reader (PerkinElmer).

### Chorioallantoic membrane assay (CAM)

CAM assay followed established procedures^28^. Initially, 4 × 10^6^ cells were seeded on embryonic day (ED) 8. By ED11, visible xenografts had formed and were treated with either a control (H_2_O; *n* = 66) or with the luciferase-expressing XVir-N-31 derivate XVir-N-1 (127.75 MOI; *n* = 67). Tumors were harvested and D-Luciferin potassium salt (2591-17-5, Sigma-Aldrich) was added at ED12 (24 hpi; *n* = 38), ED14 (72 hpi; *n* = 38) and ED16 (120 hpi; *n* = 57) to measure luminescence intensity. Xenografts were processed into FFPE-blocks for IHC staining.

### Statistical data acquisition

Statistical analyses used IBM SPSS 29.0.0.0 (SPSS, Chicago, IL, USA) and GraphPad Prism 10 (GraphPad, San Diego, CA, USA). Non-parametric Mann-Whitney U tests compared two groups, while Kruskal-Wallis and Dunn’s multiple comparison tests compared more than two groups. Descriptive Fisher’s exact tests and two-sided log-rank tests were performed in order to correlate clinico-pathological parameters. Kaplan-Meier curves and log-rank tests assessed overall survival (OS) and relapse-free survival (RFS). Following symbols indicate two-sided p-values: * *p* > 0.05, ** *p* > 0.01 and *** *p* > 0.001.

## Electronic supplementary material

Below is the link to the electronic supplementary material.

Supplementary Material 1

## Data Availability

The datasets used and/or analysed during the current study are available from the corresponding author on reasonable request.
