# Supplementary material for: Oncolytic virotherapy provides a potent therapy option for squamous bladder cancer
Source: Sci Rep. 2025 Apr 18;15:13443. doi: 10.1038/s41598-025-96419-3 (PMC12008219; doi:10.1038/s41598-025-96419-3)

**Supplementary Table S1:** Clinico-pathological parameters of patient cohort with squamous bladder cancer collected by the German Study Group of Bladder Cancer (DFBK e.V.) and analyzed in this study.

| Parameter | Categorization | sq-BLCA (n = 89) | |
| --- | --- | --- | --- |
|  |  | **[N]** | **[%]** |
| Gender | Female  Male | 46  43 | 51.7  48.3 |
| Age at diagnosis | Median  Range  30-39  40-49  50-59  60-69  70-79  80-89  90-99 | 68  34-91  3  6  16  23  28  12  1 | -  -  3.37  6.74  17.98  25.84  31.46  13.49  1.12 |
| Tumor grade | G1  G2  G3  G4  unknown | 1  25  60  1  2 | 1.12  28.09  67.42  1.12  2.25 |
| Tumor stage | pT2  pT3  pT4  unknown | 12  58  14  5 | 13.48  65.17  15.73  5.62 |
| Lymph node status | Nx  N0  N1  N2 | 17  53  12  7 | 19.10  59.55  13.48  7.87 |
| Lymphatic vessel invasion | Lx  L0  L1 | 31  18  40 | 34.83  20.22  44.94 |
| Vein invasion | Vx  V0  V1 | 35  13  41 | 39.33  46.07  14.61 |
| Residual tumor | Rx  R0  R1  unknown | 24  49  14  2 | 26.97  55.06  15.73  2.25 |

**Supplementary Table S2:** Clinico-pathological parameters in relation to YB-1 expression in DFBK sq-BLCA cohort.

|  | YB-1 expression IRS^b^ | | | | |  |
| --- | --- | --- | --- | --- | --- | --- |
|  | ***n****^a^* | **Low** | **High** | **p-value**^c^ | **Spearman r** | |
| Parameter |  |  |  |  |  | |
| Gender |  |  |  |  |  | |
| Female | 46 | 14 | 32 | 0.448 | 0.082 | |
| Male | 43 | 10 | 33 |  |  |  |
| Tumor stage^d^ |  |  |  |  |  | |
| pT2 | 12 | 3 | 9 | 1.000 | <0.001 | |
| pT3-pT4 | 72 | 18 | 54 |  |  |  |
| Grading^d^ |  |  |  |  |  | |
| G1-G2 | 26 | 12 | 14 | **0.014** | **0.268** | |
| G3-G4 | 61 | 12 | 49 |  |  |  |
| pN status^d^ |  |  |  |  |  | |
| pN negative | 53 | 14 | 39 | 0.053 | 0.230 | |
| pN positive | 19 | 1 | 18 |  |  |  |
| Age |  |  |  |  |  | |
| <68.00 | 42 | 11 | 31 | 0.730 | -0.037 | |
| ≥68,00 | 44 | 13 | 31 |  |  |  |

^a^Only bladder cancers with substantial squamous component >70% of tumor area were included; ^b^dichotomized at 25% quartile; ^c^Fisher’s exact test; ^d^According to WHO 2004 classification; Significant p-values are marked in bold face.

**Supplementary Table S3:** Primer sequences and PCR conditions

**Table S3.1:** Primer sequences for expression analyses

| Gene | Orientation | Primer sequences 5’ 🡪 3’ | T_m_ (Annealing temperature) [°C] | Length [bp] |
| --- | --- | --- | --- | --- |
| *GAPDH* | fwd  rev | GAAGGTGAAGGTCGGAGTCA  AATGAAGGGGTCATTGATGG | 60  56 | 20  20 |
| *Fiber* | fwd  rev | AAGCTAGCCCTGCAAACATCA  CCCAAGCTACCAGTGGCAGTA | 58  61 | 21  21 |
| *ß-Actin* | fwd  rev | TAAGTAGGTGCACAGTAGGTCTGA  AAAGTGCAAAGAACACGGCTAAG | 58  57 | 24  23 |
| *GATA3* | fwd  rev | AGTACAGCTCCGGACTCTTC  CCACAGTTCACACACTCCCT | 59  59 | 20  20 |
| *KRT5* | fwd  rev | CGCAACACCAAGCATGAGAT  GCTTGTTCCTGGCATCCTTG | 60  60 | 20  20 |
| *KRT6* | fwd  rev | AAGGCGTTGGACAAGTCAAC  CCAAGACCACTGCCATAGGA | 58  58 | 20  20 |

**Table S3.2:** Mastermix for qPCR

| Substances | Volume [µl] |
| --- | --- |
| SYBR-Green PCR Mix | 5 |
| Primer Mix:  10 µl Primer rev [10M]  10 µl Primer fwd [10M]  80 µl H_2_O | 0.5 |
| DNase/RNase free H_2_O | 3.5 |
| over all | **9** |

PCR-reaction volume of 10 µl over all, consisting of 9 µl mastermix and 1 µl cDNA.

**Table S3.3:** Cycle-conditions of qPCR

| Cycle | Repetitions | Time | Temperature [°C] |
| --- | --- | --- | --- |
| 1 | 1 | 30 sec | 95.0 |
| 2 | 40 | 5 sec | 95.0 |
| 3 | 40 | 30 sec | T_m_ |
| 4 | 1 | 5 sec | 65.0 |
| 5 | 1 | 0.5°C/cycle | 95.0 |
| 6 | 1 | hold | 4.0 |

**Table S3.4:** Mastermix for cDNA synthesis

| Substances | Volume [µl] |
| --- | --- |
| MgCl_2_ [25 mM] | 4 |
| Reverse transkriptase buffer [10x] | 2 |
| Oligo-dT nucleotide [500 g/l] | 1 |
| dNTP-mix [10 mM] | 2 |
| Random primer | 1 |
| AMV-reverse transcriptase | 0.6 |
| RNase-inhibitor [40 U/l] | 0.5 |

**Table S3.5:** Cycle-conditions of cDNA-synthesis

| Cycle | Time | Temperature [°C] |
| --- | --- | --- |
| 1 | 10 | 70 |
| 🡪 adding mastermix after incubation of 10 min | | |
| 2 | 10 | 25 |
| 3 | 15 | 42 |
| 4 | 5 | 95 |
| 5 | 5 | 4 |

**Supplementary Figure S1: Raw immunoblot images related to main Figure 3E.** Blue boxes highlight the relevant bands for GAPDH and YB-1 used in main Figure 3E. Red signals indicate overexposure detected by the imaging system, and exposure times were adjusted accordingly for each protein. **A)** Western blot membranes showing GAPDH (36 kDa) protein level as a loading control in the cell lines SCaBER, RT112, p-SCC, and p-UC. Exposure time: 1 minute. **B)** Western blot membranes showing YB‑1 (50 kDa) protein levels as the protein of interest in the cell lines SCaBER, RT112, p-SCC, and p-UC. Exposure time: 5 minutes.


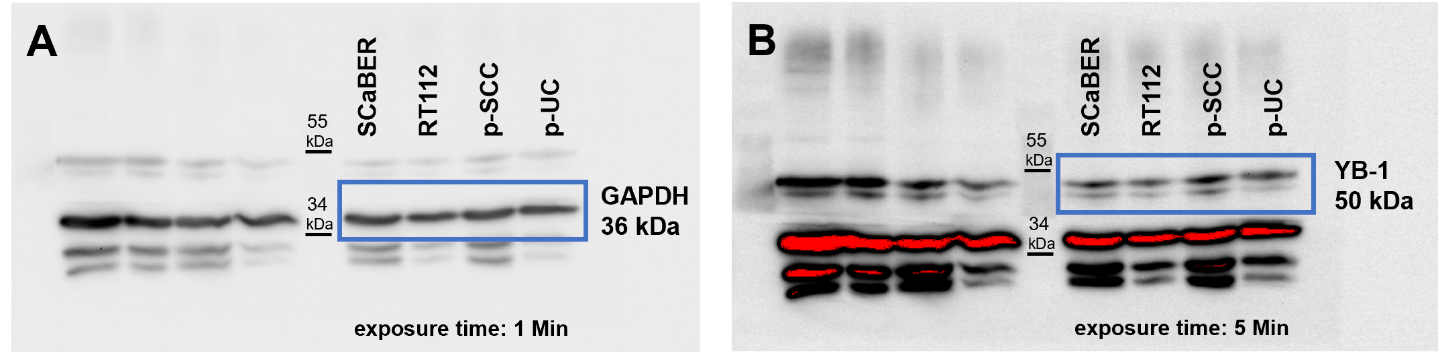

Supplement: Supplementary file 1 — Supplementary Material 1 [file 41598_2025_96419_MOESM1_ESM.docx]
